# Sleep and Mental Health among Paramedics from Australia and Saudi Arabia: A Comparison Study

**DOI:** 10.3390/clockssleep2020019

**Published:** 2020-06-08

**Authors:** Wahaj Anwar A. Khan, Russell Conduit, Gerard A. Kennedy, Ahmed Abdullah Alslamah, Mohammad Ahmad Alsuwayeh, Melinda L. Jackson

**Affiliations:** 1 Psychology Discipline, College of Science, Engineering & Health, School of Health and Biomedical Sciences, RMIT University, P.O. Box 71, Bundoora, VIC 3083, Australia; russell.conduit@rmit.edu.au (R.C.); gerard.kennedy@rmit.edu.au (G.A.K.); 2 Occupational Health Department, Faculty of Public Health and Health Informatics, Umm Al-Qura University, Makkah, P.O. Box 715, Saudi Arabia; 3 Psychology, Federation University Australia, Ballarat, VIC 3353, Australia; 4 Institute for Breathing and Sleep, Austin Health, Melbourne, VIC 3084, Australia; melinda.jackson@monash.edu; 5 General Supervisor of Emergency Medical Services Affairs, Saudi Red Crescent Authority, Riyadh 11129, Saudi Arabia; aslamah@srca.org.sa; 6 General Director of Training Department, Saudi Red Crescent Authority, Riyadh 11129, Saudi Arabia; msuwayeh@srca.org.sa; 7 Turner Institute for Brain and Mental Health, School of Psychological Sciences, Monash University, Clayton, VIC 3800, Australia

**Keywords:** depression, PTSD, paramedics, Saudi Arabia, occupational health

## Abstract

Paramedics face many challenges while on duty, one of which is working different types of shifts. Shift work has been linked to a number of health issues such as insomnia, depression, and anxiety. Besides shift work, Saudi paramedics, a group that has not been investigated for sleep or mental health issues previously, may be facing more demands than Australian paramedics due to lower numbers of paramedics in comparison to the general population. The aim of this study was to investigate the prevalence of sleep and mental health disorders among paramedics in Saudi Arabia and Australia. Paramedics were invited to complete a survey to assess stress, post-traumatic stress disorder (PTSD), depression, anxiety, daytime sleepiness, insomnia, sleep quality, shift work disorder, obstructive sleep apnoea, fatigue, and general health. A total of 104 males Saudi paramedics (*M* age = 32.5 ± 6.1 years) and 83 males paramedics from Australia (*M* age = 44.1 ± 12.1 years) responded to the survey. Significantly higher rates of depression, PTSD, insomnia, and fatigue, along with significantly poorer physical functioning were observed among Saudi paramedics in comparison with Australian paramedics. However, Australian paramedics reported significantly poorer sleep quality and general health in comparison to Saudi paramedics. After removing the effect of driving and working durations, outcomes were no longer significant. The higher burden of depression and PTSD among Saudi paramedics may be explained by longer hours spent driving and longer work durations reported by this group. Taking into consideration the outcomes reported in this study, more investigations are needed to study their possible effects on paramedics’ cognition, performance, and safety.

## 1. Introduction

The continuous demand for 24-h emergency support requires paramedics to work shift schedules that are outside the standard 8:00 a.m. to 5:00 p.m. schedule [1]. The adverse consequences of shift work have been well described in the literature, and there is now evidence to suggest that it is becoming an increasingly serious public health issue [2]. According to Zverev et al. (2009), sleep deprivation is the main outcome experienced by shift workers [3]. Sleep deprivation may contribute to the onset of many health issues, including mental health disorders [4]. In addition to shift work, paramedics have to cope with other significant challenges, including trauma, accidents, and death. This places them at higher risk of developing mental health issues. Global levels of stress and depression are high among paramedics, with the implication that the health and safety of patients and paramedics are potentially compromised [5]. More work is required to fully understand the burden of mental health issues among paramedics.

The duties, regulations and challenges that paramedics face differ from one country to another. Such variations probably contribute to the burden of mental health and sleep disorders. The comparison of different populations can be useful in informing researchers and adding useful knowledge to the field including: (1) awareness that different traditions/cultures can affect the perception of various occupational risks or hazards; and (2) the development of more comprehensive and effective control strategies that are applicable in different countries [6]. There is little known about paramedics in the Middle East, in general, and Saudi Arabia, in particular. Therefore, this study compared a Middle Eastern country where advanced rules and regulations of occupational health and safety (OHS) are emerging, to a country with well-developed OHS regulations. Such a comparison may provide unique insights about this population, along with a better understanding of commonalities in paramedics across cultures.

One of the key differences observed in current official figures regarding paramedics in Saudi Arabia and Australia is the total population size. The total number of paramedics in Saudi Arabia in 2017 was estimated to be 7864 active paramedics [7]. In Australia, the number of paramedics is double that, with 17,800 being the official estimate in 2018 [8]. The populations of Saudi Arabia (32,612,641) and Australia (25,180,200) estimated for the same year [9,10], mean that in Australia, there is one paramedic for every 1415 citizens, while in Saudi Arabia, there is one paramedic for every 4147 citizens. Thus, a single paramedic in Saudi Arabia services four times more people than a paramedic in Australia. This suggests that Saudi paramedics have a much greater workload and may face greater threats to their wellbeing than paramedics in Australia.

Another important difference between Saudi Arabia and Australia that could impact mental health and well-being is the rate of motor vehicle accidents. Saudi Arabia has one of the highest annual fatality rates for motor vehicles accidents in the world [11], with approximately 24 fatalities per 100,000 people [11]. In contrast, Australia has a motor vehicle fatality rate that is much lower at 4.6 fatalities per 100,000 people [12]. Therefore, Saudi paramedics would be dealing with more traumatic events due to higher rates of accidents, which would put them at greater risk of developing anxiety, depression and PTSD [13,14].

The aim of this study was to compare sleep and mental health problems between Saudi Arabian and Australian paramedics. We hypothesized that Saudi Arabian paramedics would report higher levels of sleep and mental health issues than Australian paramedics due to greater work load demands.

## 2. Method

### 2.1. Participants

The recruitment of paramedics for this study was facilitated and supported by the Saudi Red Crescent Authority (SRCA). Printed versions were distributed to the Saudi paramedics and electronic versions of the survey were distributed to paramedics in Australia. The study was restricted to active paramedics who worked any type of shift. A total of 100 paramedics were required to satisfy power calculations based on a moderate effect size (0.30), a significance level of *p* < 0.05 and a power of 80 [15]. A total of 121 Saudi Arabian male paramedics (only males work in this occupation in Saudi Arabia) participated in the survey from the Western region of Saudi Arabia. The response rate to the survey was 30%. In addition, the responses of 83 males from a sample of paramedics from Victoria, Australia to the same survey in a previous study (response rate = 6.8%) [16] were included in the analyses to provide a comparison with the Saudi sample.

### 2.2. Materials

The survey contained a set of validated sleep, mood, and health questionnaires and required approximately 30 min to complete. The initial part of the survey gathered demographic information, including age, gender, height, weight, use of cigarettes (smoking), medical history, use of medications, daily caffeine consumption, and a few work-related questions to determine current shift schedule, weekly working hours, weekly driving hours for work purposes, and current position in the SRCA. The validated set of surveys included in this study are listed below. A group of validated instruments to assess sleep, mood and general health were included in the survey (Table 1).

### 2.3. Procedure

The Australian survey was distributed across the state of Victoria, Australia from December 2017 until December 2018, and managed by the Ambulance Employee Australia Victoria (AEAV). A Qualtrics survey link was distributed to the Australian paramedics via emails and was also posted to their web-based newsletter. Distribution of the survey across the Makkah District, Saudi Arabia, from November 2018 until January 2019 was managed by the SRCA. Saudi paramedics were actively recruited from their work stations. Paramedics were asked to read the participants information sheet and to consent before taking the actual survey. No incentives were provided to participants. The study was approved by the Human Research Ethics Committee at the Royal Melbourne Institute of Technology (Approval # 21420).

### 2.4. Statistical Analyses

The data were analysed using the IBM Statistical Package for the Social Sciences (SPSS) version 24, Macintosh and Microsoft versions. Data distributions were adequate in terms of normality, linearity, and homoscedasticity. The data were also tested for outliers, missing values, and data entry errors. Data cleaning led to the elimination of 17 participants out of the total 121 Saudi respondents due to missing respondent data. The prevalence of sleep and mental health outcomes were presented as percentages or means and standard deviations. Independent samples *t*-tests were used to examine group differences for sleep and mental health variables. Age, BMI, driving hours and working hours were significantly different between the groups, therefore multivariate analysis of covariance (MANCOVA) was subsequently used to further investigate the unique differences between the two groups on the other variables while controlling for these demographic and work-related factors.

## 3. Results

A total of 104 male paramedics from Saudi Arabia who submitted complete data were included in the final data set. Data from Australian male paramedics (*n* = 83) that has been published previously [16] were included as a comparison to the Saudi sample. Demographics and work information for the two cohorts are presented in Table 2. The rotating shift was the most prevalent shift reported by both cohorts, with 95% of Saudi paramedics and 76% of Australian paramedics having reported working in a rotating shift, while 8% of the Australian and 5% of Saudi paramedics worked in a fixed shift. Rotating shift is a roster that includes 4 days of duty with both day and night shifts in a single roster, while the fixed shift is 4 days of duty of either day or night shifts in a single roster. The rotating roster for the Saudi paramedics consisted of two days of standard day shifts (12 h/shift) and two days of night shifts (12 h/shift). The rotating roster for the Australian paramedics consisted of two days of standard day shifts (10 h/shift) and two days of night shifts (14 h/shift). Sixteen percent of Australian paramedics worked a rural shift roster of 8 consecutive days of day shifts with overnight on-call. Saudi paramedics reported only three days of break/recovery in-between rosters compared to four days reported by Australian paramedics. The Saudi paramedics were significantly younger than the Australian paramedics (*p* < 0.001). The mean BMI was significantly higher among the Australian sample compared to the Saudi sample (*p* < 0.05). The Saudi paramedics reported significantly longer working (*p* < 0.05) and driving (*p* < 0.001) durations (hours) per week in comparison to the Australians (Table 2).

### 3.1. A Comparison between Saudi and Australian Paramedics across Sleep and Mental Health Outcomes

Table 3 shows the means and standard deviations of the Saudi and Australian paramedics for sleep and mental health variables. Saudi paramedics had significantly higher scores for stress, depression, PTSD and insomnia symptoms in comparison to Australians, while the Australian paramedics had significantly higher scores for fatigue and poorer sleep quality. The calculated effect size was large for depression symptoms, with small to medium effects for the rest of the variables. There were no significant differences in for anxiety symptoms and daytime sleepiness between the two groups of paramedics.

For questionnaires with published criteria for “at risk” scores, the prevalence for “at risk” individuals is shown in Table 4. Australian paramedics reported a greater prevalence of perceived stress, fatigue, poor sleep quality, anxiety, SWD and OSA in comparison to Saudi paramedics. However, Saudi paramedics reported higher rates of insomnia, daytime sleepiness, depression and PTSD.

MANCOVAs were conducted to control for the effects of age and BMI on the outcomes of the assessments for sleep (PSQI and ISI), mental health (BDI and PSQI-A), general health (SF-36: physical functioning, general health, and fatigue), stress (PSS), and fatigue (FSS). Saudi paramedics reported significantly higher depression (*F* (3, 114) = 6.52; *p* < 0.001; partial η2 = 0.15), PTSD (*F* (3, 114) = 9.77; *p* < 0.001; partial η2 = 0.20) and insomnia (*F* (3, 114) = 3.17; *p* < 0.05; partial η2 = 0.07) symptoms, and poorer general health (*F* (3, 114) = 9.42; *p* < 0.001; partial η2 = 0.19) than Australian paramedics. Australian paramedics reported significantly higher fatigue (*F* (3, 114) = 14.24; *p* < 0.001; partial η2 = 0.27), poorer sleep quality (*F* (3, 114) = 3.16; *p* < 0.05; partial η2 = 0.07), and lower physical functioning (*F* (3, 114) = 8.13; *p* < 0.001; partial η2 = 0.17) compared to Saudi paramedics. After controlling for the effects of age and BMI, stress; PSS (*F* (3, 114) = 2.20; *p* > 0.05; partial η2 = 0.05) and fatigue; FSS (*F* (3, 114) = 0.96; *p* > 0.05; partial η2 = 0.02) scores were no longer significantly different between two groups of paramedics.

MANCOVAs were used to control for the effect of driving and working durations on the main outcomes of the study including sleep (ISI and PSQI), mental health (BDI and PSQI-A), and fatigue (FSS). Scores were no longer significant, after controlling for driving durations, for insomnia (*F* (1, 132) = 3.56; *p* > 0.05; partial η2 = 0.02), sleep quality (*F* (1, 132) = 1.64; *p* > 0.05; partial η2 = 0.01), depression (*F* (3, 132) = 3.87; *p* > 0.05; partial η2 = 0.02), PTSD (*F* (3, 132) = 3.41; *p* > 0.05; partial η2 = 0.02), and fatigue (*F* (3, 132) = 0.43; *p* > 0.05; partial η2 = 0.01). After controlling for work duration, insomnia (*F* (1, 132) = 0.24; *p* > 0.05; partial η2 = 0.01), sleep quality (*F* (1, 132) = 0.03; *p* > 0.05; partial η2 = 0.01), depression (*F* (3, 132) = 0.50; *p* > 0.05; partial η2 = 0.01), PTSD (*F* (3, 132) = 0.80; *p* > 0.05; partial η2 = 0.01), and fatigue (*F* (3, 132) = 0.10; *p* > 0.05; partial η2 = 0.01) symptoms were no longer different between the two groups.

### 3.2. A Comparison between Saudi and Australian Paramedics across General Health Questionnaire (SF-36) Subscales

Results of the SF-36 general health questionnaire for the Saudi and Australian paramedics are shown in Table 5. Australian paramedics had a significantly higher score for physical functioning and significantly lower scores on the fatigue and general health subscales in comparison to Saudi paramedics. The calculated effect sizes for general health and fatigue scores were large. Scores on the other items of the SF-36, including role limitation due to physical health, role limitation due to emotional problems, emotional well-being, social functioning, and pain, were not significantly different between the two groups of paramedics.

## 4. Discussion

The present study investigated the prevalence of sleep and mental health concerns among Saudi paramedics and conducted a comparison with previously collected data from males in a sample of Australian paramedics [16]. Saudi paramedics reported significantly higher rates of perceived stress, depression, PTSD, and insomnia compared to paramedics in Australia. In contrast, Australian paramedics reported significantly poorer sleep quality compared to Saudi Arabian paramedics, even after controlling for age and BMI. For the general health questionnaire, Saudi paramedics reported significantly higher levels of fatigue and poorer physical functioning, but better general health, compared to Australian paramedics, with large effect sizes observed. However, it seems that driving and working durations have a significant effect on insomnia, sleep quality, depression, PTSD, and fatigue levels. After removing their effects, outcomes were no longer significant between Saudi and Australian paramedics.

Paramedics from Saudi Arabia revealed more mental health concerns than Australian paramedics, including depression and PTSD. The prevalence of self-reported depression and severity of depressive symptoms among Saudi paramedics was significantly higher compared to Australian paramedics with a large effect size observed, with similar figures to that found in a previous Australian sample [38]. The prevalence and severity of self-reported PTSD were also significantly higher among Saudi paramedics (41%) compared to prevalence rates among Australian paramedics, and prevalence rates of paramedics from other countries, including Germany and Brazil with 15% and 20%, respectively [39,40]. Moreover, perceived stress was significantly higher among Saudi paramedics compared to Australians, but more Australian paramedics screened positive in the lower end of the perceived stress scale. The severity of self-reported anxiety was not different between the two samples with a small effect size observed, with more than half of the two cohorts screening positive. Paramedics from Saudi Arabia reported higher mental health concerns than paramedics from Australia including depression, PTSD, and stress.

Saudi paramedics have, in general, not been investigated for sleep and mental health problems. However, a recent study evaluated the occupational challenges that Saudi paramedics face during active duty. It reported, from their own perception, certain barriers or challenges that paramedics reported facing, including traffic congestion, harassment from family members, lack of staff competence, lack of trust and confidence, lack of independence, resistance from patients, involvement in legal issues, and impression of the paramedics on the general community and family members of patients [41]. Such challenges may affect the mental health of the paramedics; for example, verbal abuse was significantly linked to higher rates of depression among nurses in the United States [42]. In addition, traffic congestion was found to be associated with poorer mental health outcomes among bus drivers [43]. Another contributor to an increased burden of depression among Saudi paramedics may be their extremely low population (7864 paramedics) as compared to the population of Australian paramedics (17,800) [7,8]. This is particularly represented in the longer working and driving durations reported by the Saudi paramedics, which was significantly related to the depression scores of the Saudi paramedics. One study has found a significant association between longer driving hours and poorer mental health [44]. Another recent report indicated that depressive symptoms were linked to longer working duration [45]. In addition, a five-year follow-up study reported that longer working duration is a risk factor for developing depression [46].

Saudi paramedics reported significantly higher levels of PTSD than Australian paramedics, which is an indicator of greater exposure to trauma [47]. Higher rates of fatal car accidents in Saudi Arabia compared to Australia are likely to contribute to this burden of PTSD among Saudi paramedics. The fatality rate due to car accidents is much higher in Saudi Arabia than Australia, with 24 fatalities per 100,000 people and 4.6 fatalities per 100,000 people, respectively [11,12]. Other possible causes of higher PTSD rates are lack of organizational support, lack of appropriate training, and conflict with patients’ family members [47], which have previously been reported by Saudi paramedics to be significant challenges or barriers [41]. According to Flory (2015), trauma exposure can lead to developing both PTSD and depression [48] which are both complex disorders that are more prevalent among Saudi than Australian paramedics. Possible predictors of depression and PTSD among Saudi paramedics are higher fatal accident rates, traffic congestion, lack of organizational support, lack of appropriate training, and more exposure to trauma. Most importantly, and supported by findings from the present study and previous shift work reports [44,45,46], higher work-load represented by longer driving and working durations may have a negative relationship with depression and PTSD scores of shift workers in general, and paramedics in particular.

There are many consequences of depression and PTSD to the health and safety of paramedics and patients in need of emergency medical support. Depressive symptoms are linked to impaired cognition, particularly impaired decision-making and problem-solving abilities, which are critical abilities for emergency support [49]. Greater rates of symptoms of PTSD have also been linked to impaired cognition, lowered performance, and impaired decision-making [50]. Depression and PTSD combined can result in more prevalent and more severe cognitive impairments [48]. Impaired cognition, especially during emergency medical support, is a serious issue for the safety of both the emergency services workers and the patients. More studies are required to investigate if cognitive issues are prevalent among paramedics and if they are related to mental health outcomes or not.

Sleep and fatigue were also poor among all paramedics in this study. Insomnia was significantly higher and more prevalent among Saudis compared to Australian paramedics, with a moderate effect size observed. The prevalence of excessive daytime sleepiness among Saudi paramedics, a typical sign of insomnia, was higher than Australian paramedics. However, the rate of poor sleep quality among Saudi paramedics was lower than in the Australian cohort, with over 80% of the Australian paramedics meeting the criteria for poor sleep quality. This is similar to previous reports of 68% meeting criteria for poor sleep in Australian paramedics [38]. Similarly, fatigue was less prevalent among Saudi paramedics than in Australian paramedics. The prevalence of likely OSA was higher in Australian paramedics, which could be explained by older age and the higher BMI of the Australian sample. The prevalence of SWD was particularly high in the current study compared to previous reports in firefighters from the United States (9%) [51], with over half of the Saudi and Australian paramedics at risk for this disorder. The high rates of SWD in these two samples are concerning and suggest that this occupational group may not be coping with the demands of their schedules. This may lead to higher mental health issues, as SWD was found to be a significant contributor to depression and anxiety symptoms among hospital shift workers in Australia [52]. Such variations in sleep outcomes can be influenced by many factors including age, BMI, health conditions, shift type, or work-load. This highlights the need for additional support and education about shift work, particularly in graduate paramedics who may be facing shift work for the first time.

Medical errors are serious issues that can arise from sleep problems and fatigue. For example, reducing sleepiness through reducing working hours among medical interns was linked to lower reported medical errors [53]. Sleepiness was linked to higher odds of accidents among nurses who worked on rotating shifts as compared to nurses worked in fixed shifts [54]. In addition, lower psychomotor performance was significantly related to higher sleepiness scores in nurses [55]. Moreover, higher work-place errors were strongly associated with higher levels of fatigue among medical residents [56]. Generally, shift work is linked to higher levels of fatigue and sleep disturbances, which in turn may affect workers’ performance and safety.

Although sleep disorders were common among all paramedics in this study, Saudi paramedics reported significantly higher levels of insomnia. Insomnia is one of the most important contributors to the burden of depression, especially in shift workers [57]. Even though there is little data investigating the effects of insomnia on depression among paramedics, insomnia was a significant predictor to the variance of rates and severity of depression in Australian paramedics [16]. Moreover, Courtney et al. (2013) reported a significant association between chronic fatigue and depression among Australian paramedics [58]. Other studies have focused on reporting the prevalence of depression rather than investigating the burden [38,59], but studies from other shift worker populations support the proposed relationship, such as a study of Canadian shift workers reporting a positive association between insomnia symptoms and depression [60]. Similarly, higher rates of depressive symptoms among US firefighters were explained by sleep deprivation [61]. Thus, the greater variance in rates of depression may be explained by higher rates of insomnia among Saudi paramedics.

This is the first study to investigate mental health and sleep disorders in Saudi paramedics using validated instruments. The study provided robust and detailed information for this population, and the data will act as a strong base for future studies. The current study reported the prevalence of sleep and mental health problems among Saudi paramedics and compared the results with Australian paramedics in a cross-sectional study. Statistically, age and BMI impacted the levels of perceived stress and fatigue between the two cohorts. However, it was important to report the uncontrolled findings between the two populations, with this step, including age and BMI as covariates, being exploratory. Response bias is a common issue with such a design but can be solved by conducting follow-up or prospective cohort studies. In addition, self-selection bias is another common issue that may overestimate the current findings. A comparison between rosters (rotating shift vs fixed/rural shifts) could not be done due to the fact that the majority of both samples reported working in rotating shift schedule. The difference in the response rate for the Saudi and Australian paramedics is likely due to the recruitment procedure. The survey was conducted online for the Australian paramedics, whereas the Saudi paramedics were actively recruited at work stations with printed copies. The usage of emails for work purposes is different between Saudi and Australia. So, the recruitment procedure was conducted in such a way as to make the study known to the Saudi paramedics. In addition, this study included only male paramedics from Saudi Arabia, as this occupation employs only males in Saudi Arabia, which may affect the comparison to previous findings. However, a comparison was conducted to only male paramedics from Australia. Moreover, due to data availability, the study could not specify the paramedic/citizen ratio for the studied states (Makkah District in Saudi and Victoria in Australia). Generalizing to the total population sizes was the closest available way of describing this relationship.

In conclusion, in comparison to Australian paramedics, Saudi paramedics reported significantly higher rates of depression and PTSD. This could be explained by higher rates of insomnia, longer driving durations, longer working durations, and higher rates of fatal car accidents which need to be handled by paramedics. Depression and PTSD together may affect the safety of the paramedics and the patients, so future studies must focus on investigating the cognitive functions of paramedics during duty and any effects on their performance and with regards to safety. Employing more paramedics in ambulance services and implementing fatigue risk management strategies [62] may help to improve the sleep and mental health outcomes by reducing the work-load.

## Figures and Tables

**Table 1:** Validated sleep and mental health questionnaires used in the study.

Questionnaire	Arabic Validation	Cut Off	Items	Reliability/Validity
General Health Questionnaire (SF-36) [17]	Yes [18]	NA **	36	Yes
Perceived Stress Scale [19]	Yes [20]	(>13)	14	Yes
Beck Depression Inventory-Short Form [21]	Yes [22]	(>4)	13	Yes
State-Trait Anxiety Inventory-Short Form [23]	Yes [23]	(>36)	6	Yes
Shift-work Disorder Screening Questionnaire [24]	Yes [25]	NA **	4	Yes
Pittsburgh Sleep Quality Index [26]	Yes [27]	(>5)	18	Yes
Pittsburgh Sleep Quality Index-Addendum for PTSD * [28]	Yes [29]	(>3)	10	Yes
Epworth Sleepiness Scale [30]	Yes [31]	(>10)	8	Yes
Insomnia Severity Index [32]	Yes [33]	(>14)	7	Yes
Berlin Questionnaire for OSA * [34]	Yes [35]	NA **	10	Yes
Fatigue Severity Scale [36]	Yes [37]	(>4)	9	Yes

Note. * (PTSD) post-traumatic stress disorder, (OSA) obstructive sleep apnea, ** no cut off for the SF-36 subscales, Shift-work Disorder Screening Questionnaire was calculated by a special algorithm, and Berlin Questionnaire was calculated by risk criteria where 2 or more positive categories represented individuals at high-risk.

**Table 2:** Means and standard deviations of Saudi and Australian paramedics across age, BMI, working and driving durations per week. Statistical comparisons of the groups using independent samples *t*-tests with *p* value and effect size (Cohen’s d) are shown.

	Australian Paramedics *M* (*SD*)	Saudi Paramedics *M* (*SD*)	*t (df), p, Cohen’s d.*
Age (years)	44.1 (12.1)	32.5 (6.1)	*t* (180) = −8.43, *p* < 0.001, *d* = 1.22
BMI *	27.9 (4.3)	26.1 (5.1)	*t* (184) = −2.57, *p* < 0.05, *d* = 0.38
Working (hours/week)	45.4 (10.7)	48.3 (2.5)	*t* (170) = 2.57, *p* < 0.05, *d* = 0.37
Driving ** (hours/week)	17.9 (11.2)	26.2 (18.7)	*t* (170) = 3.49, *p* < 0.001, *d* = 0.54

Note: * (BMI) body mass index, ** Ambulance driving.

**Table 3:** Means and standard deviations of Saudi and Australian paramedics across study variables.

Questionnaire	Australian Paramedics *M* (*SD*)	Saudi Paramedics *M* (*SD*)	*t (df), p, Cohen’s d.*
PSS	19.5 (4.1)	21.9 (10.3)	*t* (168) = 1.921, *p* < 0.05, *d* = 0.31
BDI-SF	7.2 (6.8)	17.4 (11.9)	*t* (166) = 6.523, *p* < 0.001, *d* = 1.05
PSQI-A	2.6 (3.2)	4.6 (4.3)	*t* (157) = 3.271, *p* < 0.05, *d* = 0.54
ISI	11.5 (5.9)	14.2 (7.1)	*t* (155) = 2.469, *p* < 0.05, *d* = 0.41
FSS	4.3 (1.3)	3.6 (1.9)	*t* (163) = −2.797, *p* < 0.05, *d* = 0.45
PSQI	8.9 (4.1)	6.6 (4.8)	*t* (157) = −3.215, *p* < 0.05, *d* = 0.53
STAI-SF	41.1 (18.5)	38.9 (12.7)	*t* (164) = −0.871, *p >* 0.05, *d* = 0.13
ESS	8.2 (6.1)	9.9 (5.1)	*t* (153) = 1.912, *p >* 0.05, *d* = 0.31

Note: Perceived Stress Scale (PSS), Beck Depression Inventory-Short Form (BDI-SF), Pittsburgh Sleep Quality Index-Addendum (PSQI-A), Insomnia Severity Index (ISI), Fatigue Severity Scale (FSS), Pittsburgh Sleep Quality Index (PSQI), State-Trait Anxiety Inventory-Short Form (STAI-SF), and Epworth Sleepiness Scale (ESS). Statistical comparisons of the groups using independent samples *t*-tests with *p* value and effect size (Cohen’s d) are shown.

**Table 4:** Incidences of Saudi and Australian paramedics across study variables.

Questionnaire	“At Risk” Criteria	Australian Paramedics	Saudi Paramedics
PSS [19]	>28	93%	31%
BDI-SF [21]	>19	32%	41%
PSQI-A [28]	>3	29%	42%
ISI [32]	>14	34%	51%
FSS [36]	>3	74%	56%
PSQI [26]	>4	81%	54%
STAI-SF [23]	>36	61%	53%
ESS [30]	>12	22%	32%
SWD * [24]	NA *	54%	51%
BQ ** [34]	NA **	46%	36%

Note: * Calculated by a special algorithm made by the original author, ** two positive groups or more indicated positive outcome, Perceived Stress Scale (PSS), Beck Depression Inventory-Short Form (BDI-SF), Pittsburgh Sleep Quality Index-Addendum (PSQI-A), Insomnia Severity Index (ISI), Fatigue Severity Scale (FSS), Pittsburgh Sleep Quality Index (PSQI), State-Trait Anxiety Inventory-Short Form (STAI-SF), Epworth Sleepiness Scale (ESS), Shift-Work Disorder (SWD), and Berlin Questionnaire (BQ) for Obstructive Sleep Apnea (OSA).

**Table 5:** Comparison of means and standard deviations for the general health questionnaire (SF-36) subscales between Saudi Arabian and Australian paramedics.

SF-36 Subscales	Australian *M* (*SD*)	Saudi *M* (*SD*)	*t (df), p, Cohens d*
Physical functioning	87.5 (15.1)	74.1 (24.2)	*t* (170) = −4.258, *p* < 0.001, *d* = 0.66
Role physical	72.8 (33.3)	71.3 (29.9)	*t* (170) = −3.00, *p >* 0.05, *d* = 0.04
Bodily pain	69.1 (21.6)	71.5 (22.8)	*t* (170) = 0.733, *p >* 0.05, *d* = 0.11
General health	52.5 (25.1)	71.6 (18.8)	*t* (170) = 5.711, *p* < 0.001, *d* = 0.86
Vitality/fatigue	44.1 (21.7)	67.9 (21.5)	*t* (170) = 7.241, *p* < 0.001, *d* = 1.10
Social functioning	72.7 (26.1)	66.6 (23.4)	*t* (170) = −1.632, *p >* 0.05, *d* = 0.24
Role emotional	63.7 (41.7)	72.4 (27.7)	*t* (170) = 1.637, *p >* 0.05, *d* = 0.24
Mental health	64.1 (20.3)	69.3 (21.4)	*t* (170) = 1.630, *p >* 0.05, *d* = 0.25
